# Cytokine responsiveness of CD8^+^ T cells is a reproducible biomarker for the clinical efficacy of dendritic cell vaccination in glioblastoma patients

**DOI:** 10.1186/2051-1426-2-10

**Published:** 2014-05-13

**Authors:** Richard G Everson, Richard M Jin, Xiaoyan Wang, Michael Safaee, Rudi Scharnweber, Dominique N Lisiero, Horacio Soto, Linda M Liau, Robert M Prins

**Affiliations:** 1 Departments of Neurosurgery, University of California Los Angeles, Los Angeles, CA 90095, USA; 2 Medicine, University of California Los Angeles, Los Angeles, CA 90095, USA; 3 Jonsson Comprehensive Cancer Center, University of California Los Angeles, Los Angeles, CA 90095, USA; 4 Brain Research Institute, David Geffen School of Medicine at UCLA, University of California Los Angeles, Los Angeles, CA 90095, USA; 5 Molecular and Medical Pharmacology, University of California Los Angeles, Los Angeles, CA 90095, USA

**Keywords:** T cells, Tumor immunity, Dendritic cells, pSTAT-5, Phospho-flow cytometry, Glioblastoma

## Abstract

**Background:**

Immunotherapeutic approaches, such as dendritic cell (DC) vaccination, have emerged as promising strategies in the treatment of glioblastoma. Despite their promise, however, the absence of objective biomarkers and/or immunological monitoring techniques to assess the clinical efficacy of immunotherapy still remains a primary limitation. To address this, we sought to identify a functional biomarker for anti-tumor immune responsiveness associated with extended survival in glioblastoma patients undergoing DC vaccination.

**Methods:**

28 patients were enrolled and treated in two different Phase 1 DC vaccination clinical trials at UCLA. To assess the anti-tumor immune response elicited by therapy, we studied the functional responsiveness of pre- and post-vaccination peripheral blood lymphocytes (PBLs) to the immunostimulatory cytokines interferon-gamma (IFN-γ) and interleukin-2 (IL-2) in 21 of these patients for whom we had adequate material. Immune responsiveness was quantified by measuring downstream phosphorylation events of the transcription factors, STAT-1 and STAT-5, via phospho-specific flow cytometry.

**Results:**

DC vaccination induced a significant decrease in the half-maximal concentration (EC-50) of IL-2 required to upregulate pSTAT-5 specifically in CD3^+^CD8^+^ T lymphocytes (p < 0.045). Extended survival was also associated with an increased per cell phosphorylation of STAT-5 in cytotoxic T-cells following IL-2 stimulation when the median post/pre pSTAT-5 ratio was used to dichotomize the patients (p = 0.0015, log-rank survival; hazard ratio = 0.1834, p = 0.018). Patients whose survival was longer than two years had a significantly greater pSTAT-5 ratio (p = 0.015), but, contrary to our expectations, a significantly lower pSTAT-1 ratio (p = 0.038).

**Conclusions:**

Our results suggest that monitoring the pSTAT signaling changes in PBL may provide a functional immune monitoring measure predictive of clinical efficacy in DC-vaccinated patients.

## Background

Immunotherapy for the treatment of cancer has begun to show clinical benefit, with several agents recently gaining FDA approval [1,2]. One such approach being studied for the treatment of glioblastoma is the administration of dendritic cells (DC), loaded with autologous tumor lysate or glioma-associated antigens, to engender an immunologic response against these tumors. Initial studies of DC-based vaccine therapy for malignant gliomas have shown acceptable safety and toxicity profiles, and multi-center randomized Phase II and III studies are currently underway [3-18]. As these and other similar agents transition to clinical use, immunomonitoring assays must be developed in order to monitor whether individual patients are responding to therapy [19], and to predict who may respond in the future.

In previous clinical trials, the majority of the immune monitoring strategies employed have not yielded an association with the clinical effects. Assays such as delayed type hypersentivity (DTH) responses, immunohistochemistry, ELISpot and measurement of cytokines produced by activated lymphocytes in blood and cell culture using ELISA, have been studied [3-5,10-12,14,20-24], but have yielded conflicting results. Many of these assays are not cell type specific, and rather, measure effects in the entire population of cells, which can dilute the activity of small populations of highly active cells. Flow cytometry can be used for the highly sensitive and precise measurement of cell type frequencies and cytokine release assays. However, there has not been a consensus set of cell surface phenotype profiles or intracellular cytokine staining approach that is consistent with vaccine-elicited immune and clinical responses [25,26]. To date, no single immune monitoring assay has consistently shown a correlation with immune response to DC vaccination or with overall survival of patients receiving DC vaccines.

Our understanding of the determinants of effector immunity as well as the agents of cancer-induced immune dysfunction continues to evolve and immune monitoring efforts should reflect these insights [27,28]. Available evidence suggests that lymphocyte responsiveness to cytokines is a key determinant in the generation and maintenance of immune effector cells [29,30]. This responsiveness has been found to be context dependent and variably impaired in cancer patients [31-36]. While this may be due to innate, individual patient-specific factors, it may also result from cancer-induced immune dysfunction and be reflective of the overall balance of proinflammatory and immune regulatory factors influencing the internal milieu, and appears especially prominent in patients with malignant glioma [27,28].

Phosphoflow, a flow cytometry-based technique to measure the phosphorylation status of key intracellular signaling molecules such as STATs, can be used to measure, on a per-cell basis, the capability of different immune cell populations to respond to cytokines [37]. The JAK-STAT signaling pathways lead to the phosphylation of STAT-1 and STAT-5, and are activated by the binding of cytokines IFN-γ and IL-2, respectively, to cytokine receptors located on the cell membrane [38]. The phosphorylated STATs (pSTAT) form dimers that trans-locate into the nucleus to initiate gene transcription programs. As a downstream product, the measurement of phosphorylated STATs serves to quantify the functional capacity of cells to respond to cytokines, which in turn should reflect whether they remain functional or have reached an anergic or tolerogenic state where they are unable to respond. Previously, our group has found evidence suggesting that the surrounding immune environment factors into the success or failure of DC vaccines with gene expression profiling, regulatory cell frequency, and the expression of negative regulatory molecules all associating with vaccine success [9,12,39]. In the present study, we analyzed the phosphorylation events of STAT-1 and STAT-5 following cytokine stimulation with IFN-γ and IL-2, respectively, in order to determine the functional responsiveness of PBL subpopulations and compared this responsiveness before and after DC vaccination. We hypothesized that changes in the functional cytokine responsiveness of lymphocyte sub-populations, pre to post DC vaccination, would convey important information regarding the effectiveness of the vaccine in a non-antigen-specific manner. Such an approach could be used as a uniform surrogate for immunotherapy. Herein, we report that DC vaccination reproducibly enhances IL-2 responsiveness in PBL, and increases in overall survival were observed in glioblastoma patients whose IL-2 responsiveness was elevated. Contrary to our expectations, we also found a negative correlation with responsiveness to IFN-γ, as measured by pSTAT-1, wherein reduced responsiveness to IFN- γ- was associated with improved survival.

## Results

### DC vaccination results in a reduction of the EC-50 for IL-2 in peripheral blood CD3^+^CD8^+^ T cells

**Figure 1:**
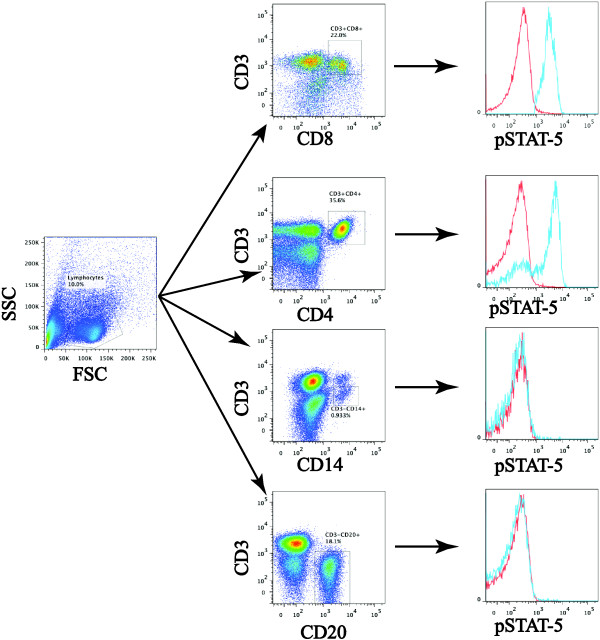
**Gating schematic for phospho-flow cytometry staining.** PBMC (pre/post DC vaccination) were stimulated with either IFN-γ (pSTAT-1; not shown) or IL-2 (pSTAT-5) for 20 minutes, followed by phospho-flow cytometry. Cells were surface labeled with antibodies to CD3, CD4, CD8, CD14, and CD20, followed by fixation, permeabilization, and intracellular staining with antibodies to pSTAT-1 and pSTAT-5. In the pSTAT-5 histograms the red tracing represents unstimulated, while the blue tracing represents IL-2 stimulated cell populations.

We hypothesized that DC vaccination would activate and expand glioma-specific T cells whose recall response to gamma chain survival cytokines and/or T_H1_-type cytokines, such as interleukin 2 (IL-2) and interferon gamma (IFN-γ), would be enhanced. To test this, differences in the functional responsiveness of PBMC subsets following DC vaccination were evaluated and compared with each patient’s overall survival using a Cox Proportional Hazard model and Kaplan-Meier survival analysis. In order to assess functional responsiveness, the dynamic levels of activated pSTAT-1 and pSTAT-5 within PBMCs was monitored following a short 20-minute ex vivo IFN-γ or IL-2 stimulation, respectively. Phospho-specific flow cytometric analysis of patient PBMCs was quantified in the following subsets of PBMCs: Monocytes (CD3^-^CD14^+^), Helper T-Cells (CD3^+^CD4^+^), Cytotoxic T-Cells (CD3^+^CD8^+^), and B-Cells (CD3^-^CD20^+^) (Figure 1). Inherent heterogeneity in PBMC compositions and intracellular pSTAT populations between patients was controlled for by generating post to pre DC vaccination ratios for every leukocyte population in each patient.

**Figure 2:**
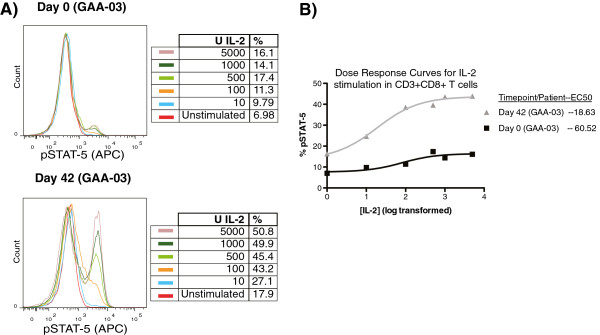
**Dose-dependent changes in pSTAT-5 by CD3**^**+**^**CD8**^**+ **^**T cells in response to IL-2.** PBMC (pre/post DC vaccination) were stimulated with graded doses of IL-2 for 20 minutes, followed by intracellular staining for pSTAT-5. **(A)** Representative example of the pSTAT-5 dose response staining from patient #GAA-03 before (top) and after (bottom) DC vaccination. **(B)** Non-linear plot comparing the dose response of CD3 + CD8+ T lymphocytes before and after DC vaccination in patient #GAA-03. The calculated EC-50 values are listed.

Stimulation of PBMC subsets with IL-2 resulted in a dose-dependent increase in both the percentage (%) and median fluorescence intensity (MFI) of pSTAT-5 in CD3^+^CD4^+^ and CD3^+^CD8^+^ T lymphocytes (Figure 2A). Such a dose-dependent relationship was not prevalent for pSTAT-1 in response to IFN-γ stimulation (data not shown). We also evaluated whether the half-maximal effective concentration (EC-50) of IL-2 changed after DC vaccination, and if changes in the EC-50 of IL-2 correlated with patient survival. In 8 out of 9 patients, from whom we had sufficient cells for complete dose response stimulations, a reduction in the EC-50 for IL-2 was observed in CD3^+^CD8^+^ T cells after DC vaccination, but not in CD3^+^CD4^+^ T cells (Figure 2B, Table 1). We also observed an increase in the percentage of CD3 + CD8+ T cells expressing pSTAT-5, at a given dose of IL-2 stimulation, after DC vaccination in selected patients (Figure 2), mirroring findings from our pre-clinical model [40]. When the ratio of each patient’s EC-50 for IL-2 (post/pre DC vaccination) was analyzed using Cox Regression, a significant association with patient survival was found (hazard ratio = 2.21; 95% CI (1.11, 4.43); p = 0.024). These results strongly suggest that DC vaccination is specifically followed by a heightened response to a typical gamma chain cytokine associated with lymphocyte survival in cytotoxic T cells from glioblastoma patients.

**Table 1:** IL-2 responsiveness in peripheral blood T cells before and after DC vaccination

**Patient ID**	**Overall survival (months)**	**Pre-DC Vax CD3+CD8+ EC-50**	**Post-DC Vax CD3+CD8+ EC-50**	**Pre-DC Vax CD3+CD4+ EC50**	**Post-DC Vax CD3+CD4+ EC-50**
1-708	33.83	128.2	13.16	3.528	13.39
34-730	16.23	126.2	66.01	20.73	0.005
36-852	>43.9	27.95	0.017	4.407	0.002
6-815	17.3	1.854	0.196	0.002	2.355
19-539	34.9	8.223	14.27	1.386	1.178
GAA-03	35.3	60.52	18.63	29.64	0.003
GAA-05	11.73	38.2	34.69	11.43	5.245
GAA-07	17.2	16.52	6.07	5.446	5.237
GAA-09	11.0	31.19	22.38	7.2	7.7
AVERAGE:		50.70	19.15	9.31	3.90
SEM:		16.90	6.38	3.10	1.30
T-test:		0.045		0.223	

^1^The EC-50 for IL-2 responsiveness (pSTAT-5) was calculated in CD3^+^8^+^ and CD3^+^CD4^+^ peripheral blood T cells after a 20-minute stimulation with increasing dosages of IL-2, FACS analysis, and log transformation.

### Changes in CD8^+^ T-Cell responsiveness to IL-2 correlates with survival after DC vaccination in glioblastoma patients

**Figure 3:**
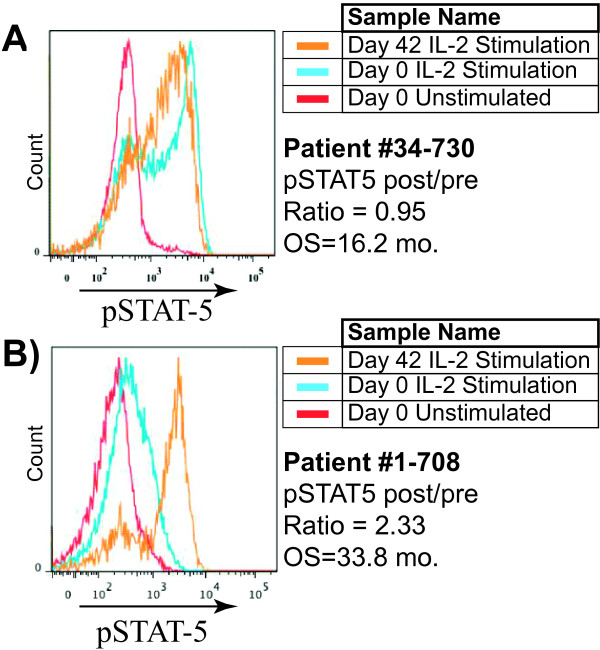
**T cell responses to STAT-5 are associated with overall survival in glioblastoma patients treated with DC vaccination.** PBMC (pre/post DC vaccination) were either left unstimulated or stimulated with IL-2 for 20 minutes, followed by phospho-flow cytometry. pSTAT-5 histograms were gated from CD3^+^CD8^+^ T cells. Representative histograms from patients #34-730 **(A)** and #1-708 **(B)**, document the pSTAT-5 expression in PBL samples when unstimulated, or stimulated with 500 IU IL-2 (pre/post DC vaccination). Note the differences in the pattern of pSTAT-5 expression before and after DC vaccination.

A Cox proportional hazards model was also utilized to investigate the correlation between the functional responsiveness of PBL to a single, fixed dose of cytokine stimulation and patient survival. Because this design required much fewer PBL, we were able to study the peripheral responses of 20/28 glioblastoma patients treated with DC vaccination. Comparison of MFI fold-change ratios in CD3^-^CD14^+^ Monocytes, CD3^+^CD4^+^ Helper T-cells, and CD3^-^CD20^+^ B-cell populations (post/pre DC vaccination) with patient survival yielded no statistically significant correlations. However, a statistically significant positive correlation was observed between the CD3^+^CD8^+^pSTAT5^+^% and MFI (post DCVax/pre DCVax ratio) after a single dose of IL-2 stimulation and overall patient survival (Figure 3A-B; hazard ratio = 0.1834; 95% CI = 0.045, 0.749; p = 0.018). As is apparent in Figure 3, CD3 + CD8+ T cells whose responsiveness to IL-2 was elevated after DC vaccination compared with before DC vaccination (Figure 3B) had signficantly longer overall survival than patients whose CD3 + CD8+ T cell responsiveness to IL-2 did not change (Figure 3A). Based on this correlation, a one unit increase in the ratio of the pSTAT-5 ratio (post to pre DC vaccination) from IL-2-stimulated cytotoxic T-cells following DC vaccination reduces the risk of death in vaccinated patients by 5.45 times.

**Figure 4:**
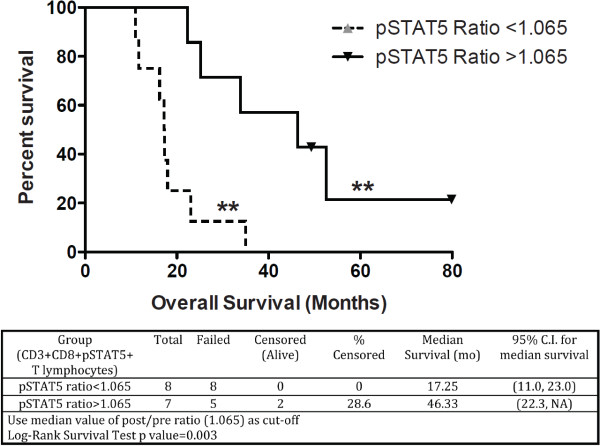
**Predictive immune monitoring using pSTAT-5 expression in peripheral blood lymphocytes from glioblastoma patients treated with DC vaccination.** A recursive partitioning survival tree was calculated from the median pSTAT-5 ratio in CD3^+^CD8^+^ T lymphocytes to dichotomize patient subsets. Kaplan-Meier survival is plotted.

A recursive partitioning survival tree was also used to dichotomize the pSTAT-5 post/pre DC vaccination ratio in CD8^+^ T-cells. This model was implemented as a means to develop a functional immune monitoring measure of immunological responses predictive of clinical efficacy in DC-vaccinated patients. To stratify patients in an unbiased fashion, we chose the median pSTAT-5 ratio (post/pre DC vaccination) as a cut-off, which effectively segregated patients whose pSTAT-5 ratio increased (>1) or decreased (<1) after treatment. When the median pSTAT-5 ratio (post/pre DC vaccination) in CD3^+^CD8^+^ T cells was used to dichotomize patients, a highly significant difference in survival was observed (p = 0.003, Log-rank test). The median survival below this cut-off value was 17.25 months (95% C.I. (11.0, 23.0) and 46.3 months (95% C.I. (22.3, NA)) above the cut-off value (Figure 4). Therefore, the development of elevated pSTAT-5 expression in CD3^+^CD8^+^ T cells after DC vaccination is associated with dramatically extended survival in glioblastoma patients.

### Reciprocal pSTAT responsiveness in CD8^+^ T lymphocytes after DC vaccination correlates with survival in glioblastoma patients

**Figure 5:**
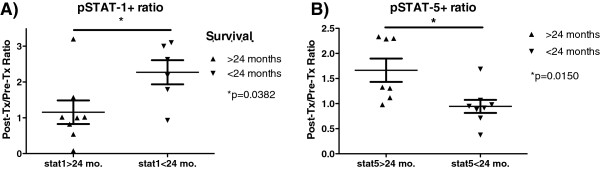
**pSTAT-5 and pSTAT-1 expression in CD3**^**+**^**CD8**^**+ **^**T cells effectively segregates glioblastoma patients after DC vaccination.** The post-Tx/pre-Tx ratio of stimulated pSTAT-5 **(A)** or pSTAT-1 **(B)** MFI were compared with patients whose overall survival was less than or greater than 2 years. *p = 0.015 for pSTAT-5, *p = 0.0382 for pSTAT-1 (2-tailed T-test).

2-year survival is known to be a significant endpoint for glioblastoma patients who receive standard-of-care treatment [41-44]. Based on our analysis of pSTAT-1 and pSTAT-5 expression in PBL before, and after DC vaccination, we stratified glioblastoma patients based on 2-year survival. We then examined the phosporylation of STAT-1 and STAT-5 from CD3^+^CD8^+^ T cells. A significant difference was found between the ratio (post/pre vaccination) of pSTAT-1 and pSTAT-5 when stratifed by 2-year survival (Figure 5). Patients with overall survival greater than 2 years had a significantly greater pSTAT-5 ratio (p = 0.015), but significantly lower pSTAT-1 ratio (p < 0.038). Therefore, reciprocal responses to IL-2 and IFN-γ after DC vaccination are also associated with one of the primary survival endpoints in glioblastoma.

## Discussion

Our findings provide evidence that alterations in cytokine responsiveness of PBLs from glioblastoma patients treated with dendritic cell vaccination are associated with overall survival and may be reflective of vaccine efficacy. We measured STAT phosphorylation in the PBLs of glioblastoma patients enrolled in clinical trials of autologous tumor lysate loaded or tumor antigen associated peptide pulsed dendritic cell vaccine. Initial measurements were taken from PBLs before the patient had received the DC vaccine. Comparison measurements were made on PBL from patients after three biweekly DC vaccinations were given. When ratios of cytokine responsiveness were compared in an individual pre- and post-vaccination, we found a strong correlation that enhanced responsiveness of CD8^+^ T lymphocytes to IL-2 was associated with long-term survival of greater than 2 years. DC vaccination reproducibly induced elevated IL-2 (pSTAT-5) responsiveness, the magnitude of which was also directly associated with survival. An increased responsiveness to IFN-γ (pSTAT-1) at the 6-week post-vaccine time point compared to pre-vaccination, on the other hand, was unexpectedly associated with worsened prognosis. We believe such information, if validated, could be used to help determine if a patient was successfully responding to immunotherapy with DC vaccination.

This phospho-flow immune monitoring study is the first, to our knowledge, to assess the effectiveness of a clinical immunotherapy trial in glioblastoma patients using a functional technique not based on antigen-specific T responses. These studies stem directly from pre-clinical results in which pro-inflammatory cytokine production was shown to enhance the expansion and survival of glioma-specific CD8^+^ T cells after DC vaccination, in a STAT5-dependent fashion [40,45]. As yet, it is unclear exactly which subset of CD4^+^ or CD8^+^ T lymphocytes may be directly influenced by DC vaccination. Our pre-clinical studies suggest that local pro-inflammatory cytokine production after DC vaccination enhanced the generation of effector memory CD8^+^ T lymphocyte populations (CD44^hi^, Eomes^low^, CCR7^low^), but with evidence of a lymph node homing phenotype and central memory cell markers (CD62L^+^, CD49d^+^) [40]. Our clinical immune monitoring design did not allow us to additionally determine whether distinct T lymphocyte subsets were sensitized to STAT-5 signaling, but we are currently designing new antibody cocktails that will allow for such simultaneous data acquisition (CD45RO, CD62L, CCR7) in our ongoing clinical trials. We believe that such an immune monitoring tool is critical for a vaccine comprised of DC pulsed with autologous tumor lysate because each patient’s array of tumor-associated antigens will be distinct, and thus the identity of each patient’s tumor-specific T cells will additionally be different. We also believe that such an immune monitoring strategy will also be important in the future because meta-analyses have demonstrated that vaccination with whole-tumor antigens induced higher objective clinical responses than vaccination with defined tumor antigens [46]. The recent clinical success of negative costimulatory molecule blockade in patients of diverse solid tumor histology [47,48] suggests that immune monitoring of non-antigen-specific lymphocyte function could also disclose relevant information on the endogenous anti-tumor immune response.

In addition to being a surrogate marker that may indicate a successful DC vaccination, the levels of cytokine responsiveness before and after vaccination may shed insight into the cellular mechanisms that are important for a generating an effective long-term immune response. Active immunotherapies, such as dendritic cell vaccines, depend on provoking a host immune response, the ultimate goal of which is the generation of potent, long-lasting tumor-specific T lymphocytes. Such T cells are thought to develop against many tumors, but are ultimately defeated by the immunosuppressive effects of the tumors themselves, which secrete anti-inflammatory cytokines both systemically and into the tumor microenvironment and activate immune-regulatory cell populations. The major signaling pathway induced by IL-2 is through phosphorylation of STAT-5. When the post:pre ratio of pSTAT-5 was examined, it was clear that patients who demonstrated improved survival had ratios greater than 1 and developed a reduced EC-50 to IL-2. Our hypothesis is that immune suppression from the brain tumor acts as an antagonist to IL-2 signaling in T cells, which is counteracted by the DC vaccine. Biologically, this suggests that the CD8^+^ lymphocytes either became more responsive to IL-2 (e.g., increased potency) or expressed elevated levels of pSTAT-5 after vaccination. We have previously shown that enhanced sensitivity to IL-2 signaling and frequency of pSTAT-5 regulates the clinical responsiveness of IL12-primed CD8+ T cells to intracranial tumors [40]. STAT-5 signaling also plays a crucial role in normal immune function. STAT-5 is required for IL-2-induced cell cycle progression in T cells and is required for antigen-induced T-cell recruitment into tumor tissue [49]. Mice deficient in STAT5 exhibit alterations in NK cell function and decreased T-cell and B-cell proliferation in response to chemokines [50]. Thus, increased pSTAT-5 responsiveness to IL-2 may suggest that the DC vaccine has overcome the immunosuppressive effects of the tumor and effectively primed glioma-specific T cells.

The major signaling pathway induced by IFN-γ is through the phosphorylation of STAT-1. IFN-γ is a well-characterized molecule and plays a central role in the immune response. Many assays assessing cellular immune function do so by measuring the release of IFN-γ in response to a stimulus, such as antigen recognition by T cells. Therefore, we initially expected that the detection of elevated pSTAT-1 expression would be beneficial. However, we observed the opposite to be true; in patients that demonstrated an increased post:pre pSTAT1 ratio, overall survival tended to be worse. There are several possible explanations for this, which mostly center on the chronicity of the IFN-γ signaling. Recent studies indicate that IFN-γ has pleiotropic effects on the immune system, with different effects during primary and secondary responses to an antigen [51]. At first stimulatory, over time, continued exposure to IFN-γ can lead to feedback inhibition in order to limit excessive tissue destruction and autoimmune disease. The main mechanisms behind these immunoregulatory roles of IFN-γ have included its ability to promote activation and function of regulatory T cells through DC-mediated immunosuppression via induction of tryptophan catabolizing enzyme IDO, iNOS, and HO-1 [52]. Therefore, increased IFN-γ signaling after DC vaccination may actually indicate that immune responses initially created by the DC vaccination are being actively suppressed/tolerized when measured 6 weeks later.

## Conclusions

A statistically significant relationship was found between the levels of cytokine responsiveness, pre and post vaccination, and overall survival in glioblastoma patients on clinical trials of DC immunotherapy. These data suggest that increased ratios of IL-2 responsiveness/pSTAT5 signaling and decreased ratios of IFN-γ responsiveness/pSTAT1 signaling may be associated with better prognosis in this patient population. We believe that cytokine responsiveness may be a novel immune monitoring measure to evaluate the effectiveness of the anti-tumor immune response in a non-antigen-specific fashion. Such a technique could be utilized to assess the function of a number of peripheral blood leukocyte populations before and after immunotherapy in cancer patients.

## Methods

### Patient eligibility

#### *Patient characteristics*

Twenty-eight patients with histologically diagnosed glioblastoma (WHO Grade IV) were enrolled into one of two Phase 1 clinical trials at UCLA investigating the use of autologous tumor lysate (ATL; UCLA IRB #03-04-053, FDA IND #11053, clinical trial registration #NCT00068510; n = 23)) or glioma-associated antigen (GAA)-pulsed (UCLA IRB #06-01-052, FDA IND #12966, clinical trial registration #NCT00612001; n = 5) DC vaccination. This study examined the 21 of 28 eligible patients for which there was adequate patient material. Of these evaluable patients, 17 patients underwent ATL pulsed DC immunotherapy, while four underwent GAA peptide-pulsed DC immunotherapy between 2003 and 2010. The demographics of patients receiving either treatment modality included 15 males and 5 females, ranging from 26-70 years old. The clinical characteristics of these patients, with multi-variate clinical comparisons, have been published previously [18]. Patient inclusion/exclusion criteria for these studies can be found at ClinicalTrials.gov (http://clinicaltrials.gov/). All patients received three biweekly vaccinations with autologous DC after surgical resection for tumor removal. All PBL used in these analyses were isolated from patients at day 0, prior to the first DC vaccination, and at day 42, two weeks after the third DC vaccination. During the six weeks of this DC vaccination treatment course, and as a requisite for trial eligibility, all patients were free of any concomitant medications known to affect immune cell function (e.g., chemotherapy, corticosteroids). All patients provided written informed consent according to UCLA Medical Internal Review Board (IRB) guidelines before treatment. Informed consent was approved by the UCLA Medical IRB and given by patients for their experimental treatment, storage of clinical data in a secured database, and research performed on residual patient tissues.

### Collection and storage of PBMC for immune monitoring

PBMCs were obtained from the peripheral blood of patient subjects in compliance with UCLA Medical IRB protocols. Peripheral blood was drawn from patients at various time points both pre and post DC vaccination. PBMCs from the peripheral blood were collected via Ficoll extraction and subsequently placed in a freezing media of 10% DMSO and 90% serum for storage in liquid nitrogen.

### Cytokine stimulation of normal PBMC and patient PBMC

Normal PBMC and patient PBMC (pre and post DC vaccination) samples were thawed at 37°C, resuspended in X-Vivo culture media and rested overnight. Following the overnight rest, cells were enumerated and resuspended in a volume of X-Vivo with 0.1% B-mercaptoethanol and 2% FBS to obtain 1x10^6^ cells/mL. Cells were then stimulated with IL-2 or IFN-γ. Each aliquot received a different treatment of cytokine, either IFN-γ, IL-2, or no stimulation. IFN-γ stimulated aliquots received 0.1 ng, 1 ng, 10 ng, or 100 ng IFN-γ. IL-2 stimulated aliquots received 100 U, 500 U, 1000 U, or 5000 U IL2. Once the cytokines had been added, the cells were incubated at 37°C for 20 minutes.

### Intracellular antibody staining

Following cytokine stimulation, the cells were fixed with 100 uL of 16% paraformaldehyde for a final concentration of 1.6% paraformaldehyde and incubated for 10 minutes at room temperature. After incubation, the cells were then washed with 2 mL of staining media, resuspended at 10^7^ cells/mL in staining media, and transferred to new tubes and stained accordingly. Among the 10 normal PBMC samples were seven single colored controls including CD3 FITC, CD14 Pacific Blue, CD4 Alexa Fluor 647, CD3 APC-Cy7, CD4 PE, CD20 PC-5.5, and CD8 PE-Cy7. The three additional normal PBMC samples were used to create normal stained and unstained cytokine unstimulated controls and a “color -2” control (containing all single color control stains excluding the FITC and AF647-conjugated antibodies). For the remaining patient PBMC samples, an antibody cocktail was created in order to ensure consistent staining of patient PBMCs. The antibody cocktail was composed of pSTAT-1 FITC, pSTAT-5 AF647, CD3 APC-Cy7, CD4 PE-Cy7, CD8 PB, CD14 PE, and CD20 PerCP-Cy5.5. The samples were subsequently incubated for 30 minutes at room temperature away from light.

### Flow cytometric analysis

Experimental data was collected via multi-color flow cytometry of the stained PBMCs using the BD LSRII flow cytometer and BD FACS diva software. In calibrating the flow cytometer, only the seven colors of interest were selected. The negative control was then analyzed and FSC and SSC voltages were modified in order to optimize the visibility of the lymphocyte population. Once properly gated, the single colored compensation controls were acquired and the voltages were modified in order to place the peak of the stained cells at 10^4^. Each single color control was gated in relative to the negative unstained control and all compensation controls were applied. Afterwards, each experimental sample was acquired at 200,000 events per sample.

### Statistical analysis

The raw data was aggregated and tabulated for analysis and study using GraphPad software v5.03 (GraphPad Software, La Jolla, CA). Descriptive statistics such as mean and standard deviation were used to summarize continuous variables, while count and percentage were used for categorical variables. Bivariate comparisons of continuous variables and categorical variables were performed using unpaired t-tests. The Kaplan Meier method and log-rank test were used to summarize the overall survival and time to progression of both trials. Univariate and multivariate Cox proportional hazards regression models were used to correlate the individual immune monitoring variables with overall survival. Recursive partitioning survival trees were built to obtain the cut-off values utilized to dichotomize each immune monitoring variable, which could differentiate the overall survival between the variables. EC-50 curves for IL-2 were generated in a standard fashion using 4-5 log dilutions of stimulatory cytokines. The log of the dose vs. response were found to follow a symmetrical sigmoidal shape and plotted using GraphPad Prism. The EC-50 was calculated using the equation: Y = bottom + (top-bottom)/(1 + 10^(log EC50-x)). The calculated EC-50 is the concentration of dose (IL-2) that elicits a response halfway between bottom and top. For all statistical investigations, tests for significance were two tailed, with a statistically significant p-value threshold of 0.05. Statistical analyses were performed using SAS 9.2 (SAS institute, Cary, NC).

## Abbreviations

DC: Dendritic cell; STAT: Signal transducer and activator of transcription; pSTAT: phosphorylated STAT; CTL: Cytotoxic T lymphocyte; DTH: Delayed type hypersensitivity.

## Competing interests

The authors declare that they have no competing interests.

## Authors’ contributions

RGE, RMJ, DNL, MS, RS, and HS performed the research, participated in the design of the studies, and helped draft the manuscript. XW performed the statistical analysis and helped draft the manuscript. LML and RMP conceived of the study, participated in the design and coordination of the study, and helped draft the manuscript. All authors read and approved the final manuscript.
